# The Effect of Regulating Zoo Visitor-Penguin Interactions on Zoo Visitor Attitudes

**DOI:** 10.3389/fpsyg.2019.02351

**Published:** 2019-10-17

**Authors:** Samantha J. Chiew, Paul H. Hemsworth, Sally L. Sherwen, Vicky Melfi, Grahame J. Coleman

**Affiliations:** ^1^Animal Welfare Science Centre, Faculty of Veterinary and Agricultural Sciences, The University of Melbourne, North Melbourne, VIC, Australia; ^2^Department of Wildlife Conservation and Science, Zoos Victoria, Parkville, VIC, Australia; ^3^Hartpury University and Hartpury College, Gloucester, United Kingdom

**Keywords:** visitor attitudes, visitor-animal interactions, zoos, little penguins, penguin behavior, exhibit manipulations

## Abstract

Understanding visitor attitudes toward zoo animals can inform the way zoos manage visitor-animal interactions by identifying the factors that may influence visitors and the way visitors interact with animals. Consequently, we investigated the relationship between visitor attitudes and penguin behavior and the effects of regulating visitor-penguin interactions on visitor attitudes and experience. Visitor attitudes toward little penguins (*Eudyptula minor*), their welfare, enclosure, visitor effects, enclosure manipulations and visitor experience at an Australian zoo were assessed. A 2 × 2 fully randomized factorial design was used to examine potential factors that may influence visitor attitudes: (1) Viewing proximity of visitors to the enclosure: “Normal viewing distance” and “Increased viewing distance” (using a physical barrier set up 2 m from the enclosure) and (2) Intensity of visitor behaviors: “Unregulated visitor behavior” and “Regulated visitor behavior” (using signage and researcher in zoo uniform). Visitor attitudes were assessed using an anonymous attitude questionnaire. Visitors were approached after they had finished viewing the penguins and were given two options to complete the questionnaire, either on an iPad on site during their zoo visit or online (URL sent via email) after their zoo visit. A total of 495 surveys (48% during zoo visit, 52% after zoo visit) were completed. Majority of respondents were non-zoo members, females and aged between 26 and 35 years old. Results revealed a significant relationship (*p* < 0.05) between little penguin behavior and visitor attitudes where the more visible, active and close penguins were to the visitor viewing area, the more positive several visitor attitude scales were. In contrast, there were only a few treatment effects of regulating visitor viewing proximity and behavior on visitor attitudes in which attitudes toward “Positive penguin characteristics” (*p* = 0.024), “Neutral visitor effects” (*p* = 0.0023) and “Physical barriers” (*p* = 0.013) were affected. This suggests that physical barriers and/or signage are factors that influence visitor attitudes. However, it is unclear if the treatment effects influenced visitor attitudes directly, or if it was the changes in penguin behavior as a consequence of the treatments that were associated with visitor attitudes. These findings have increased our understanding of the multifaceted nature of visitor attitudes and have identified some influencing factors on attitudes that can be used to inform the way zoos manage visitor-penguin interactions, but clearly further research is required.

## Introduction

Understanding zoo visitor perceptions and attitudes has been of growing importance because of the varying effects zoo visitors can have on the behavior and welfare of zoo animals (Hosey, 2013; Sherwen and Hemsworth, 2019). In particular, understanding visitor attitudes toward zoo animals can inform the way zoos manage visitor-animal interactions by identifying the factors that may influence visitors and the way they interact with animals. However, this has yet to be established and thoroughly investigated within zoos (Fernandez et al., 2009; Hosey, 2013; Sherwen and Hemsworth, 2019). Before proceeding further, it is important to define what we mean by “perceptions” and “attitudes” as these two terms are often used interchangeably. On the one hand, while closely related to attitudes, perceptions refer to an individual’s interpretation of specific situations, stimuli or objects into something meaningful to them based on past experiences (Pickens, 2005). On the other hand, attitudes refers to the “mindset or tendency to act in a certain way” where we are trying to understand or explain an individual’s behavior (Pickens, 2005). Therefore, attitudes are reflective of a positive or negative assessment of a given object which are derived from beliefs (Ajzen, 1991; Eagly and Chaiken, 1993) and are a strong determinant of behavior (Ballantyne and Parker, 2005).

Research on zoo visitors has shown that there are a variety of factors that influence visitor perceptions of zoos, zoo animals, visitor experience, viewing times and interests including exhibit design and animal characteristics such as animal size, color, activity and rarity (Rhoads and Goldsworthy, 1979; Bitgood et al., 1988; Finlay et al., 1988; Reade and Waran, 1996; Nakamichi, 2007; Margulis and Westhus, 2008; Kutska, 2009; Whitworth, 2012; Mun et al., 2013). However, despite this growing research and evidence of visitor effects on zoo animals, we have limited understanding of visitor attitudes toward specific zoo species, what influences these attitudes and how these attitudes affect visitor behavior and the way visitors interact with zoo animals (Fernandez et al., 2009; Hosey, 2013; Sherwen and Hemsworth, 2019). Understanding visitor attitudes toward specific zoo species and the factors, such as animal behavior, that may influence these attitudes, are important because of the potential implications they can have on the way zoos manage visitor-animal interactions. Research on zoo visitor-animal interactions has shown that these interactions can affect both zoo animal welfare and visitor experience and thus, visitor perceptions of zoos and zoo animals (Sherwen and Hemsworth, 2019). Negative visitor perceptions can adversely impact the mission of zoos of providing high standards of animal welfare and positive visitor experiences to support zoos as zoo-based conservation organizations (Ward and Sherwen, 2018; Sherwen and Hemsworth, 2019). Consequently, it is vital for zoos to not only understand how visitors affect zoo animals but also visitor attitudes toward specific zoo species and how potential factors such as zoo animal behavior may affect visitor attitudes. Through this understanding, zoos can then target these attitudes to potentially modify visitor behavior toward zoo animals to better manage visitor-animal interactions. However, limited research has been conducted to understand this relationship between visitor attitudes and zoo animal behavior.

Godinez et al. (2013) is one of the few studies that has investigated the influence of zoo animal behavior on both visitor behavior and visitor perceptions of the animal. They found that crowd size and visitor length of stay increased when jaguars were visible regardless of whether animals were active (e.g., eating, walking), inactive (sitting or lying down) or engaged in stereotypic behaviors (e.g., pacing and circling) compared to when “out of sight” (Godinez et al., 2013). However, visitor perceptions of the jaguars’ wellbeing were reduced when the jaguars were displaying stereotypic behaviors (Godinez et al., 2013). This study highlights how animal behavior can influence visitor perceptions, but it remains unclear whether animal behavior influences visitor behaviors as no comparisons were made between active, inactive and stereotypic behaviors on visitor dwell time. Also, Miller (2012) found that after viewing a short video of a tiger engaged in pacing behavior compared to a tiger resting, people’s perception of the level of care for the tigers at the facility decreased as did their interest in supporting zoos. It is evident from these studies, that there is a need for more robust research investigating how animal behavior affects visitor attitudes toward zoo animals and subsequently visitor behaviors. Ideally, an experimental approach should be taken whereby the interactions between visitors and animals are manipulated. Doing so, allows for causal conclusions to be drawn which enables rigorous interpretation of the effects of manipulating visitor-animal interactions on visitors and zoo animals (Cochran and Cox, 1957). Only a handful of studies thus far have applied this type of experimental approach to study zoo visitor-animal interactions (e.g., Sherwen et al., 2014, 2015a,b; Learmonth et al., 2018; Chiew et al., 2019). For example, Saiyed et al. (2019) found that zoo-housed African penguins (*Spheniscus demersus*) entering a close encounter with visitors in their enclosure in which visitors were instructed to sit quietly on a bench, showed no subsequent changes in affiliative and aggressive behaviors in comparison to no close encounter. While Sherwen et al. (2015b) and Chiew et al. (2019) found that close visitor contact markedly affected huddling, vigilance, pool use, proximity to the visitor viewing area and preening behavior of little penguins (*Eudyptula minor*) which suggests that visitors looming over penguins rather than sitting may be more fear-provoking. This type of research can help inform the way zoos manage visitor-animal interactions and may require, for example, alterations in exhibit design or the development of interventions to optimize both animal welfare and visitor attitudes and experience. Consequently, it is also important to evaluate the effects of interventions or management strategies that may be used to manage these interactions on visitors and animals.

Some studies have found that modification of zoo visitor-animal interactions using interventions or manipulations in the exhibit area such as visual or physical barriers, may affect visitor experience and potentially visitor attitudes despite the improvement in animal welfare. For example, the presence of a one-way visual screen that reduced the visibility of visitors resulted in reductions in intragroup aggression and fecal glucocorticoid concentrations in black-capped capuchins (*Sapajus apella*) (Sherwen et al., 2015a). This indicated an improvement in capuchin welfare, but was found to reduce visitor numbers at the exhibit, perhaps because of the reduced interaction with the capuchins and in turn potentially reduced visitor experience and interest in the exhibit (Sherwen et al., 2015a). Also, Chiew et al. (2019) found that regulating visitor viewing proximity and the intensity of visitor behaviors by using a physical barrier to increase visitor viewing distance by 2 m away from the enclosure, reduced little penguin fear responses toward visitors. This was indicated by a reduction in the frequency of potentially threatening visitor behaviors such as banging on enclosure features, looming over the pool and sudden movement which reduced the proportion of penguins huddling and vigilant and increased the proportion of penguin close to the visitor viewing area, surface swimming and preening in the water when the physical barrier was in place (Chiew et al., 2019). However, the physical barrier was found to reduce visitor numbers, similar to that of Sherwen et al. (2015a). In contrast, Blaney and Wells (2004) found that when camouflage netting was installed to the viewing area of a gorilla exhibit that reduced the visibility of visitors, it not only improved gorilla welfare but also improved visitor perceptions of the gorillas. Consequently, assessing visitor attitudes toward such interventions and management strategies is important so that zoos can balance animal welfare and visitor experience and feasibly manage visitor-animal interactions.

Our present study was conducted in conjunction with that of Chiew et al. (2019). Our aims were to examine the relationships between visitor attitudes and experience and penguin behavior and determine the effects of regulating visitor viewing proximity and behavior on visitor attitudes and experience.

## Methodology

**FIGURE 1:**
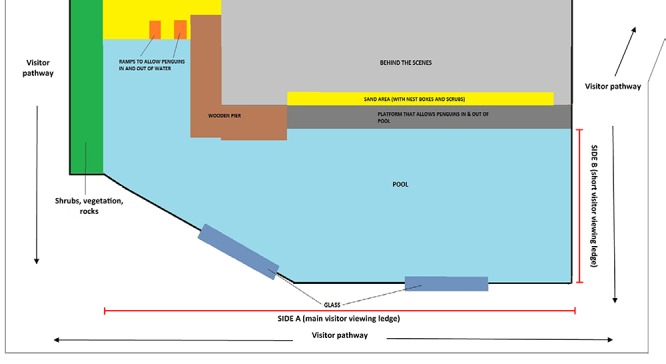
Diagram of Melbourne Zoo penguin exhibit obtained from Chiew et al. (2019).

Visitor attitudes toward little penguins were studied in conjunction with our study that investigated the effects of regulating visitor viewing proximity and the intensity of visitor behaviors on little penguin behavior and stress physiology (Chiew et al., 2019). Thus, this present study was conducted using the same methodology as Chiew et al. (2019) at the Melbourne Zoo little penguin (*Eudyptula minor*) exhibit (Zoos Victoria, Australia) which housed a breeding group of 15 little penguins in an outdoor, naturalistic 330 m^2^ enclosure consisting of sand and vegetation areas, and a large swimming pool that went up to 3 m in depth (Figure 1). The enclosure walls were 1.2 m in height and the visitor path ran along three sides of the enclosure in which the main penguin viewing positions were along the length of the pool, side A, but opportunities to view penguins also occurred on the short ledge of the pool, side B (Figure 1). The penguins were fed twice a day (9:00 and 15:30 h) and husbandry followed normal routines and remained consistent throughout the course of the study (Chiew et al., 2019).

### Design and Treatments

A 2 × 2 factorial treatment arrangement was used to examine the combined effects of regulating both visitor viewing proximity and intensity of visitor behavior on penguins (Chiew et al., 2019) and visitor attitudes and experience. The factors that were examined were as follows (Chiew et al., 2019):

(1) **Viewing proximity of visitors to enclosure** at 2 levels:
  (a) “*Increased viewing distance*” – a barrier was set up 2 m from the enclosure to increase the distance between visitors and the enclosure. This allowed unrestricted viewing of the enclosure but was a strong impediment to visitors physically interacting with the glass windows at the pool, pool water and other enclosure features.
  (b) “*Normal viewing distance*” – no barrier was in place and visitors could approach to the edge of the pool (i.e., visitors could approach within 2 m of the enclosure).
(2) **Intensity of visitor behaviors** at 2 levels:
  (a) “*Unregulated visitor behavior*” – visitor behaviors were uncontrolled.
  (b) “*Regulated visitor behavior*” – the objective of this treatment was to attempt to reduce the intensity of visitor behavior using signs requesting visitors to be quiet, move slowly in the exhibit area and avoid physically interacting with the penguins. Also, for this treatment, the researcher was dressed in zoo uniform.

Thus, there were four treatments in a factorial design as described in Table 1. For further details and description of the design and treatments imposed refer to Chiew et al. (2019).

**TABLE 1:** The 2 × 2 factorial treatment arrangement used to examine the combined effects of visitor viewing proximity (using a physical barrier to push visitors 2 m back from the enclosure) and the intensity of visitor behavior (using signage to attempt to regulate visitor behavior) on penguins and visitor attitudes and experience.

**FACTORS**		**Intensity of visitor behaviors**	
		*Unregulated visitor behavior*	*Regulated visitor behavior*
**Visitor viewing Proximity**	*Normal viewing distance*	No physical barrier and no signs (Control)	No physical barrier but signs present (Signs)
	*Increased viewing distance*	Physical barrier present but no signs (Physical barrier)	Physical barrier and signs present (Physical barrier and Signs)

Using a fully-randomized factorial design, treatments were randomly imposed for 2-day periods, two treatments per week with one day break in between (Mon-Tues and Thurs-Fri) and three replicates of each treatment (total of 24 study days). The study was conducted from the end of February to May 2016 (Summer/Autumn) over 9 weeks and was only conducted on school working days, to avoid the normal systematic variation in visitor numbers that occurs on weekends and during school holiday periods (Chiew et al., 2019). Two out of the 9 weeks had treatments with no day break in between which was due to public holidays occurring on the Monday one week and Friday the other week (Chiew et al., 2019).

The main penguin behavior measurements used from Chiew et al. (2019) for the present study were the behavioral states of the penguins including the proportion of penguins visible, huddling, distance from the visitor viewing area (sides A and B; Figure 1), resting, idle, locomoting on land, vigilant, surface swimming and diving. For further information on the behavioral sampling procedure refer to Chiew et al. (2019).

### Visitor Questionnaires

This study received Human Ethics approval from the Veterinary and Agricultural Sciences Human Ethics Advisory Group (Ethics Application 1545739.1). Questionnaires were developed and refined based on focus groups discussions with visitors at Melbourne Zoo (Melbourne, Australia) and Taronga Zoo (Sydney, Australia).

Visitors were randomly approached by student volunteers and interns (from the Animal Welfare Science Centre, University of Melbourne) during seven 30 min blocks between 09:30 and 15:15 h, after they had finished viewing the penguins and had exited the exhibit area. Visitors were asked to complete an anonymous questionnaire which assessed their attitudes toward the welfare of little penguins, the quality of their exhibit, exhibit manipulations and visitor experience. Visitors were given two options to complete the questionnaire, either on an iPad (on site during their zoo visit) or online (URL sent via email, after their zoo visit). Questionnaires were conducted on all study days.

Questionnaires took no longer than 10 min to complete and were divided into five sections: Section 1 collected information on the participants’ demographics; Section 2 collected information on the participants’ attitudes toward the little penguins and included questions such as “Do you think the little penguins are aggressive?,” “Do you think the penguins are happy?” and “On a scale from 1 to 10, how would you rate the welfare of the little penguins”; Section 3 collected information on the participants’ attitudes toward the little penguin enclosure and included questions such as “Do you think the penguin enclosure is well maintained?” and “On a scale from 1 to 10, how would you rate the little penguin enclosure?”; Section 4: collected information on the participants’ experience where for example, questions in this section included “It was exciting to see the little penguins.” and “On a scale from 1 to 10, how would you rate your experience at the penguin enclosure?”; and the final section assessed the participants’ attitudes toward manipulations to the little penguin enclosure which included questions such as “Having one-way visual barriers where penguins cannot see visitors but visitors can see penguins improves penguin welfare.” For attitude questions, a 5-point Likert scale was used which consisted of the following options: (1) Strongly disagree, (2) Disagree, (3) Neither agree nor disagree, (4) Agree, and (5) Strongly agree. The responses were scored so that disagreement with a statement had lower scores and agreements had higher scores. For rating questions, visitors were asked to rate, out of 10, the welfare of the little penguins, the little penguin enclosure and visitor experience at the enclosure where 1 was very poor and 10 was excellent.

A plain language statement was also visible at the enclosure and areas in which student volunteers and interns were located. The purpose of the plain language statement, which is a requirement for human ethics approval by the university, was to provide visitors with details and the purpose of the survey.

### Data Analysis

Statistical analyses of questionnaire responses comprised frequency distributions of demographic factors across response categories and principal component analyses (PCAs) on attitudinal data using SPSS version 25. PCAs were conducted on the attitudinal data from the questionnaire to reduce the large number of attitude variables to a relatively small number of components, where the components reflected commonalties amongst those individual variables that correlated highly with each other. Subjective labeling of each component based on semantic content of the items was performed. Cronbach’s alphas (α) were performed to measure the internal consistency of the items/questions within each component extracted from PCA (i.e., how closely related a set of items were as a group) as a measure of scale reliability. Scale mean scores for each component were calculated so that the averages were on the same scale as the original items/questions i.e., Likert scale from 1 to 5. Scale mean scores were then used as dependent variables for subsequent statistical analyses including one-way analysis of variance (ANOVA) to identify differences between treatments on scale mean scores. Prior to these ANOVAs, Levene’s test statistic was used to test for homogeneity of variance.

The data on the effects of regulating visitor viewing proximity and behavior on penguin behavior from Chiew et al. (2019), were obtained at the same time as questionnaire responses were collected. Pearson correlations were used to examine the relationship between visitor attitudes and penguin behavior where scale mean scores for each day for visitor attitudes and the angular transformed data per day for penguin behavior were used i.e., the proportion of penguins performing each behavior per day (%). This transformation was used so that the residual variation was similar in all treatments and average scale mean scores were calculated per day as penguin behavior was averaged per day. It should be noted that the penguin behavior in Chiew et al. (2019) was averaged across the 2-day period for each treatment whereas single day averages were used in the present study because different visitors were surveyed each day.

## Results

### Demographics and Percentage of Respondents

A total of 495 visitors completed the questionnaire and 639 visitors refused to complete the questionnaire; 238 were completed onsite (48%) during their zoo visit while 257 were completed online after their zoo visit (52%). Most participants were visitors living in Australia, non-zoo members and primarily females (Table 2). Also, majority of respondents were pet owners or had previously owned a pet and were aged between 26 and 35 years old (Table 2). Furthermore, most participants’ highest level of education was a university or higher education institution degree (Table 2). There was a fairly even spread of participants across the four main study treatments, however the “Physical barrier” treatment which increased visitor viewing distance from the penguin enclosure by 2 m to regulate visitor viewing proximity, had the highest percentage of surveys completed on those days (Table 2).

**TABLE 2:** Demographic information on visitors who completed the questionnaire.

**Demographic Factor**	**Control (standard zoo conditions)**	**Physical barrier**	**Signs**	**Physical barrier and Signs**	**Total**
Number of participants	114 (23.0%)	167 (33.7%)	91 (18.4%)	123 (24.8%)	495
**Residence**					
Living in Australia	87	127	82	96	392(80.0%)
Overseas	26	38	9	25	98(20.0%)
**Type of visitor**					
Zoo member	54	77	42	48	221(44.6%)
Non-zoo member	60	90	49	75	274(55.4%)
**Gender**					
Male	30	51	29	39	149(30.0%)
Female	84	116	61	84	345(69.8%)
**Previously owned/Currently own a pet**					
Yes	104	157	85	117	463(93.5%)
No	10	10	6	6	32(6.5%)
**Age**					
18–25	30	40	14	35	119(24.2%)
26–35	29	51	31	33	144(29.3%)
36–45	30	45	23	27	125(25.4%)
46–55	6	9	7	9	31(6.3%)
55+	19	21	15	18	73(14.8%)
**Highest Level of Education**					
No formal schooling	0	0	0	0	0(0%)
Primary school	0	0	1	0	1(0.2%)
Secondary school	19	27	17	27	90(18.2%)
Technical or further education institution (including TAFE College)	21	32	19	18	90(18.2%)
University or other higher education institution	74	108	53	74	309(62.4%)
Other educational institution	0	0	1	4	5(1.0%)

### Principal Component Analyses (PCA)

There were 46 attitudinal statements that were subjected to PCA (Table 3). Scale reliabilities were measured using Cronbach’s α coefficients with an α ≥ 0.70 as the criterion for acceptable reliability (Pallant, 2007). Items were included in a scale if their loading on the relevant component exceeded 0.33 (Pallant, 2007; Tabachnick and Fidell, 2007) and if, based on face validity and semantic content, they could be summarized by just one construct. Varimax or oblimin rotations were performed on component solutions of more than one factor to provide the best simple structure and to simplify interpretation (Pallant, 2007). Selection of a varimax or oblimin rotation was also determined by examining the “component correlation matrix.” If the correlations between components in this matrix were greater than 0.30, which indicates there is more than 10% overlap in variance between the components and therefore suggests they are correlated (Pallant, 2007; Brown, 2009), an oblimin rotation was used. If the correlations in the “component correlation matrix” did not exceed 0.30, then a varimax rotation was performed.

**TABLE 3:** Extracted attitude components from performing PCAs with the corresponding survey questions that loaded on each component, their loadings and scale mean scores (±standard error of mean, SEM) based on a 5-point Likert scale (1 = strongly disagree to 5 = strongly agree).

**Extracted attitude components**	**Items**	**Loadings**	**Scale mean scores ± SEM**
Positive little penguin characteristics (Cronbach’s α = 0.85)	Do you think little penguins are Playful?	0.80	3.71 ± 0.03 (*n* = 473)
	Do you think little penguins are Curious?	0.78	
	Do you think little penguins are Intelligent?	0.75	
	Do you think little penguins are Interactive?	0.75	
	Do you think little penguins are Proactive?	0.71	
	Do you think little penguins are Friendly?	0.70	
	Do you think little penguins are Social?	0.60	
Negative penguin welfare (Cronbach’s α = 0.84)	Do you think the penguins are Frightened?	0.88	2.66 ± 0.03 (*n* = 467)
	Do you think the penguins are Stressed?	0.85	
	Do you think the penguins are Frustrated?	0.73	
	Do you think the penguins are Anxious?	0.68	
	Do you think the penguins are Subdued?	0.66	
	Do you think the penguins are Bored?	0.56	
	Do you think the penguins are Under-stimulated?	0.47	
Positive penguin welfare (Cronbach’s α = 0.78)	Do you think the penguins are Alert?	0.89	3.70 ± 0.03 (*n* = 466)
	Do you think the penguins are Healthy?	0.82	
	Do you think the penguins are Happy?	0.74	
	Do you think the penguins are Expressing natural behaviors?	0.62	
	Do you think the little penguins are Calm?	0.40	
Positive visitor effects (Cronbach’s α = 0.78)	Do you think penguins find visitors entertaining?	0.89	2.82 ± 0.03 (*n* = 474)
	Do you think penguins find visitors interesting?	0.88	
	Do you think penguins find visitors novel?	0.72	
Neutral visitor effects (Cronbach’s α = 0.59)	Do you think penguins find visitors NOT fear-provoking?	0.86	3.21 ± 0.03 (*n* = 472)
	Do you think penguins are unbothered by visitors?	0.82	
Positive enclosure features (Cronbach’s α = 0.87)	Do you think the penguin enclosure is interesting to look at?	0.85	3.39 ± 0.03 (*n* = 477)
	Do you think the penguin enclosure is well maintained?	0.84	
	Do you think the penguin enclosure is natural looking?	0.82	
	The exhibit was engaging.	0.68	
	Do you think the penguin enclosure is meeting the needs of penguins?	0.54	
	Do you think the penguin enclosure is NOT bland?	0.43	
Negative enclosure features (Cronbach’s α = 0.87)	Do you think the penguin enclosure is small?	0.93	2.92 ± 0.04 (*n* = 481)
	Do you think the penguin enclosure is restrictive?	0.91	
	Do you think the penguin enclosure is NOT spacious?	0.71	
	Do you think the penguin enclosure is bland?	0.45	
	Do you think the penguin enclosure is NOT meeting the needs of penguins?	0.34	
Learning (Cronbach’s α = 0.88)	I learnt about a penguin’s natural lifestyle.	0.89	2.79 ± 0.04 (*n* = 479)
	I learnt about penguin behavior when I was at the penguin exhibit.	0.88	
	I learnt about conservation issues related to penguins.	0.87	
Experience (Cronbach’s α = 0.81)	I like being close to the penguins.	0.81	3.96 ± 0.03 (*n* = 482)
	I like seeing the penguins active and engaging in lots of behaviors.	0.78	
	It was exciting to see the little penguins.	0.77	
	It was entertaining to watch the little penguins.	0.74	
Interests (Cronbach’s α = 0.45)	I wish there was more information about the penguins at the exhibit.	0.83	3.77 ± 0.03 (*n* = 477)
	If I could, I would like to do something to help care for little penguins in captivity and in the wild.	0.75	
Visual barriers (Cronbach’s α = 0.76)	Having one-way visual barriers where penguins cannot see visitors, but visitors can see penguins improves penguin welfare.	0.90	3.68 ± 0.04 (*n* = 481)
	Having one-way visual barriers where penguins cannot see visitors, but visitors can see penguins improves visitor experience.	0.88	
Physical barriers (Cronbach’s α = 0.52)	Having physical barriers that reduce the proximity between visitors and penguins improves visitor experience.	0.92	3.47 ± 0.03 (*n* = 478)
	Having physical barriers that reduce the proximity between visitors and penguins improves penguin welfare.	0.69	

The greater the loading, the more the variables are a pure measure of the factor/component; loadings above 0.70 are considered strong/excellent (Tabachnick and Fidell, 2007). Table 3 provides the extracted attitude components, the questions that loaded on each component and the scale mean scores for each component where the higher the mean score, the more agreement and therefore more positive the attitude. Cronbach’s α coefficients are also presented in Table 3.

#### Attitudes Toward Little Penguin

A total of seven attitude questions were subjected to PCA. The Kaiser-Meyer-Olkin value was 0.86 and Bartlett’s test of sphericity was significant (*p* < 0.05). No rotation was performed as only a single component was extracted with eigenvalues exceeding 1. The component explained a total of 53.3% of the variance. Based on an inspection of the loadings, the component was labeled as “*Positive penguin characteristics*” (Table 3). Single questions related to little penguin aggressiveness and timidness were analyzed separately as they were found to not reliably measure the same underlying construct when subjected to PCA.

#### Attitudes Toward Little Penguin Welfare

A total of 12 attitude questions were subjected to PCA. The Kaiser-Meyer-Olkin value was 0.88 and Bartlett’s test of sphericity was significant (*p* < 0.05). The PCA extracted two main components with eigenvalues exceeding 1. The two components explained a total of 54.0% of the variance; component 1 explained 42.0% and component 2 explained 12.0% of the variance. An oblimin rotation was used and the two components had a correlation of −0.49. Based on an inspection of the loadings observed in the Pattern matrix, component 1 was labeled “*Negative penguin welfare*” and component 2 labeled “*Positive penguin welfare*” (Table 3). Also, the question where visitors were asked to rate the welfare of the little penguins (out of 10) was analyzed separately as it was on a different rating scale to the attitudinal statements. Overall, visitors rated little penguin welfare on average as 7.60 out of 10 (minimum = 3, maximum = 10).

#### Attitudes Toward the Visitor Effect

A total of five attitude questions were subjected to PCA. The Kaiser-Meyer-Olkin value was 0.60 and Bartlett’s test of sphericity was significant (*p* < 0.05). The PCA extracted only two main components with eigenvalues exceeding 1. A varimax rotation was used. The two components explained a total of 70.5% of the variance; component 1 explained 43.5% and component 2 explained 27.0% of the variance.

Based on an inspection of the loadings, component 1 was labeled “*Positive visitor effects*” and component 2 was labeled “*Neutral visitor effects*” (Table 3). Cronbach’s α for “*Neutral visitor effects*” was 0.59 which was below the criterion of 0.70 (Table 3). This was influenced by only two items loading on this component but the Cronbach’s α was deemed adequate based on the item loadings being above 0.70 and this component explained 27.0% of the variance.

#### Attitudes Toward the Little Penguin Enclosure

A total of nine attitude questions were subjected to PCA. The Kaiser-Meyer-Olkin value was 0.89 and Bartlett’s test of sphericity was significant (*p* < 0.05). The PCA extracted two main components with eigenvalues exceeding 1. An oblimin rotation was used in which the components had a correlation of −0.55. The two components explained a total of 67.8% of the variance; component 1 explained 56.1% and component 2 explained 11.7% of the variance. Based on an inspection of the loadings, component 1 was labeled “*Positive enclosure features*” and component 2 “*Negative enclosure features*” (Table 3). Also, a question where visitors were asked to rate the little penguin enclosure (out of 10) was analyzed separately as it was on a different rating scale to the attitudinal statements. Overall, visitors rated the little penguin enclosure on average as 6.91 out of 10 (minimum = 1, maximum = 10).

#### Attitudes Toward Visitor Experience

A total of nine attitude questions were subjected to PCA. The Kaiser-Meyer-Olkin value was 0.78 and Bartlett’s test of sphericity was significant (*p* < 0.05). The PCA extracted three components with eigenvalues exceeding 1. A varimax rotation was used. The three components explained a total of 71.4% of the variance; component 1 explained 38.8% and components 2 and 3 explained 20.9 and 11.8% of the variance, respectively.

Based on an inspection of the loadings, component 1 was labeled “*Learning*,” component 2 “*Experience*” and component 3 “*Interests*” (Table 3). Cronbach’s α for “*Interests*” was 0.45 which was below the criterion of 0.70 (Table 3). This was influenced by only two items loading on this component but the Cronbach’s α was deemed adequate because both item loadings were above 0.70 and this component explained 11.8% of the variance. Also, a question where visitors were asked to rate their experience (out of 10) at the little penguin enclosure was analyzed separately as it was on a different rating scale to the attitudinal statements. Overall, visitors rated their experience at the little penguin enclosure on average as 6.45 out of 10 (minimum = 1, maximum = 10).

#### Attitudes Toward Exhibit Manipulations

A total of four attitude questions were subjected to PCA. The Kaiser-Meyer-Olkin value was 0.59 and Bartlett’s test of sphericity was significant (*p* < 0.05). Although, the scree plot indicated only one eigenvalue exceeding 1, two components with an oblimin rotation provided a more interpretable result. The two components had a correlation of 0.32 and explained a total of 74.9% of the variance; component 1 explained 50.8%, and component 2 explained 24.0%.

Based on an inspection of the loadings, component 1 was labeled “*Visual barriers*” and component 2 was labeled “*Physical barriers*” (Table 3). Cronbach’s α for “*Physical barriers*” was 0.52 which was below the criterion of 0.70 (Table 3). This was influenced by only two items loading on this component but the Cronbach’s α was deemed adequate because the item loadings were above 0.70 and the variance explained was 24.0% for this component.

### Relationship Between Little Penguin Behavior and Visitor Attitudes

Little penguin behavior was found to be significantly correlated (*p* < 0.05) with all attitude scale mean scores, except for “Perceived Aggressiveness” and “Interests” (Table 4). The majority of the correlations fell within the moderate range, 0.40–0.59, with a few in the strong range, 0.60–0.79 (Table 4; Evans, 1996).

**TABLE 4:** Pearson correlations between scale mean scores and little penguin behavior.

	**Penguins visible**	**Huddling**	**<1 m from side A of the visitor viewing area**	**>1 m from side A of the visitor viewing area**	**<1 m from side B of the visitor viewing area**	**>1 m from side B of the visitor viewing area**	**Resting**	**Idle**	**Locomotion**	**Vigilant**	**Surface Swimming**	**Diving**
**Scale mean scores (Likert scale 1–5: 1 = strongly disagree, 5 = strongly agree)**												
Positive penguin characteristics	0.35	–0.21	0.52^∗∗^	–0.13	0.56^∗∗^	–0.23	–0.20	−0.43^∗^	–0.28	0.15	0.66^∗∗^	0.66^∗∗^
Perceived Aggressiveness	0.11	–0.16	0.08	0.13	0.05	0.11	–0.18	0.02	0.25	0.21	0.13	–0.07
Perceived Timidness	–0.15	0.03	–0.21	–0.22	–0.15	–0.22	–0.59^∗∗^	0.25	0.26	–0.12	–0.30	–0.19
Negative penguin welfare	−0.48^∗^	0.10	–0.33	0.09	−0.41^∗^	0.22	0.12	0.38	0.45^∗^	–0.20	−0.51^∗^	−0.59^∗^
Positive penguin welfare	0.44^∗^	–0.06	0.30	–0.10	0.37	–0.19	–0.32	−0.42^∗^	–0.34	0.27	0.55^∗∗^	0.59^∗∗^
Positive visitor effect	0.19	–0.22	0.27	–0.08	0.35	–0.19	–0.30	–0.31	–0.03	0.05	0.46^∗^	0.47^∗^
Neutral visitor effect	0.54^∗∗^	0.06	0.20	0.04	0.33	–0.11	–0.20	–0.26	–0.37	0.27	0.41^∗^	0.42^∗^
Positive enclosure characteristics	0.42^∗^	–0.11	0.15	–0.13	0.32	–0.28	–0.22	–0.37	−0.48^∗^	0.18	0.42^∗^	0.44^∗^
Negative enclosure characteristics	–0.13	0.23	–0.28	0.29	−0.41^∗^	0.41^∗^	0.30	0.37	0.32	0.01	−0.45^∗^	−0.44^∗^
Learning	–0.15	–0.28	0.25	−0.42^∗^	0.32	−0.50^∗^	–0.17	–0.29	–0.32	–0.37	0.29	0.39
Experience	0.30	–0.24	0.35	–0.14	0.43^∗^	–0.26	–0.11	−0.47^∗^	–0.32	0.06	0.58^∗∗^	0.65^∗∗^
Interests	–0.25	0.16	–0.27	–0.03	–0.18	–0.06	–0.02	0.23	–0.04	–0.33	–0.24	–0.27
Visual barriers	−0.50^∗^	–0.30	0.17	–0.24	0.04	–0.15	0.11	–0.03	0.39	–0.15	–0.02	–0.27
Physical barriers	−0.42^∗^	–0.62^∗∗^	0.49^∗^	−0.48^∗^	0.44^∗^	−0.48^∗^	−0.45^∗^	–0.23	0.31	–0.29	0.46^∗^	0.31
**Rating questions (scale 1–10, 1 = very poor, 10 = excellent)**												
Welfare of little penguins	0.08	–0.39	0.42^∗^	–0.34	0.50^∗^	−0.46^∗^	–0.12	–0.58^∗∗^	–0.33	–0.12	0.61^∗∗^	0.64^∗∗^
Little penguin enclosure	0.05	–0.28	0.35	−0.42^∗^	0.48^∗^	−0.55^∗^	–0.22	−0.47^∗^	−0.46^∗^	–0.07	0.48^∗^	0.38
Visitor experience at the little penguin enclosure	0.40	–0.02	0.15	–0.04	0.23	–0.14	0.01	–0.35	−0.42^∗^	0.151	0.36	0.48^∗^

*The angular transformation was used for penguin behavior. ^∗^Correlation is significant at the 0.05 level (2-tailed). ^∗∗^Correlation is significant at the 0.01 level (2-tailed).*

“Positive penguin characteristics” were positively correlated with penguins close to the visitor viewing area, surface swimming and diving and negatively correlated with penguins idle (Table 4). This was also observed for “Experience” (Table 4). Similarly, “Positive penguin welfare” was positively correlated with the proportion of penguin visible, surface swimming and diving and negatively correlated with the proportion of penguins idle. This was also found for “Positive enclosure characteristics” which was also negatively correlated with the proportion of penguins locomoting (Table 4). In contrast, “Negative penguin welfare” were negatively correlated with proportion of penguins visible, close to the visitor viewing area, surface swimming and diving and positively correlated with penguins locomoting which was also observed for “Negative enclosure characteristics” (Table 4). Furthermore, “Physical barriers” was negatively correlated with the proportion of penguins visible and huddling and positively correlated with penguins being close to the visitor viewing area and surface swimming (Table 4). When visitors were asked to rate (out of 10) the welfare of the little penguins and their enclosure, both were positively correlated with the proportion of penguins close to the visitor viewing area, surface swimming and diving and negatively correlated with the proportion of penguins idle (Table 4).

### Treatment Effects on Visitor Attitudes and Rating Questions

Analysis of variance revealed few differences in visitor attitudes (3 out of 17) between the treatment groups (Table 5). The treatment groups were: standard zoo conditions (Control), a physical barrier in place to regulate visitor viewing proximity but no signs (Physical barrier), signs present to attempt to regulate the intensity of visitor behaviors but no physical barrier (Signs) and both a physical barrier in place and signs present to regulate both visitor viewing proximity and behavior (Physical barrier and Signs; Tables 1 and 5). It was found that the treatment groups only significantly differed (*p* < 0.05) in their attitudes toward “Positive penguin characteristics” (*F*_3__,__469_ = 3.18, *p* = 0.024), “Neutral visitor effects” (*F*_3__,__468_ = 4.89, *p* = 0.0023) and “Physical barriers” (*F*_3__,__474_ = 3.64, *p* = 0.013; Table 5).

**TABLE 5:** The effect of the treatments on scale mean scores (±SEM) and rating questions.

	**Control (standard zoo conditions)**	**Physical barrier**	**Signs**	**Physical barrier and Signs**	***P*-value**
**Scale mean scores (Likert scale 1–5: 1 = strongly disagree, 5 = strongly agree)**					
Positive penguin characteristics	3.71 ± 0.05	3.81 ± 0.05	3.57 ± 0.07	3.67 ± 0.06	**0.024**
Perceived Aggressiveness	1.71 ± 0.08	1.74 ± 0.07	1.95 ± 0.11	1.92 ± 0.08	0.072
Perceived Timidness	3.56 ± 0.09	3.60 ± 0.07	3.57 ± 0.11	3.37 ± 0.08	0.17
Negative penguin welfare	2.56 ± 0.06	2.67 ± 0.05	2.76 ± 0.06	2.66 ± 0.05	0.17
Positive penguin welfare	3.76 ± 0.06	3.74 ± 0.04	3.64 ± 0.06	3.64 ± 0.05	0.24
Positive visitor effect	2.81 ± 0.06	2.82 ± 0.06	2.78 ± 0.07	2.83 ± 0.06	0.96
Neutral visitor effect	3.37 ± 0.07	3.28 ± 0.06	2.99 ± 0.08	3.15 ± 0.07	**0.0023**
Positive enclosure characteristics	3.42 ± 0.08	3.39 ± 0.06	3.31 ± 0.08	3.42 ± 0.06	0.69
Negative enclosure characteristics	2.88 ± 0.08	2.95 ± 0.07	3.04 ± 0.09	2.85 ± 0.07	0.44
Learning	2.71 ± 0.09	2.87 ± 0.07	2.76 ± 0.09	2.76 ± 0.09	0.49
Experience	3.98 ± 0.07	3.93 ± 0.05	3.95 ± 0.06	3.97 ± 0.05	0.88
Interests	3.78 ± 0.06	3.77 ± 0.05	3.77 ± 0.07	3.76 ± 0.06	0.99
Visual barriers	3.50 ± 0.08	3.74 ± 0.06	3.76 ± 0.08	3.70 ± 0.07	0.061
Physical barriers	3.26 ± 0.07	3.52 ± 0.06	3.55 ± 0.09	3.54 ± 0.07	**0.013**
**Rating Questions (scale 1–10, 1 = very poor, 10 = excellent)**					
Welfare of little penguins	7.52 ± 0.17	7.57 ± 0.14	7.46 ± 0.20	7.81 ± 0.15	0.47
Little penguin enclosure	7.04 ± 0.21	6.78 ± 0.18	6.57 ± 0.25	7.21 ± 0.19	0.16
Visitor experience at the little penguin enclosure	6.65 ± 0.21	6.42 ± 0.18	6.33 ± 0.25	6.38 ± 0.21	0.74

*P-values less than 0.05 are in bold.*

A “Least Significant Difference” *post hoc* test was performed and found that attitudes toward “Positive penguin characteristics” differed between visitors in the “Physical barrier” and “Signs” treatment groups: visitors exposed to the physical barrier had more positive attitudes compared to visitors only exposed to signs. In other words, visitors exposed to the physical barrier agreed more that the little penguins were playful, curious, intelligent, interactive, proactive, friendly and social compared to visitors exposed to signs (Table 5).

For attitudes toward “Neutral visitor effects,” differences were found between visitors in the “Control” group and “Signs” treatment group and between visitors in the “Control” and “Physical barrier and Signs” treatment group (Table 5). Visitors in the “Control” agreed more that penguins do not find visitors fear-provoking and are unbothered by visitors compared to visitors that were only exposed to signs or both to a physical barrier and signs (Table 5). Also, differences were found between visitors in the “Physical barrier” and “Signs” treatment groups where visitors only exposed to a physical barrier agreed more that penguins do not find visitors fear-provoking and are unbothered by visitors compared to visitors exposed only to signs, who on average neither agreed nor disagreed visitors affect penguins (Table 5).

Attitudes toward “Physical barriers” differed between visitors in the “Control” and visitors in all other treatment groups (Table 5). Visitors in the “Control” had fairly neutral attitudes (i.e., neither agreed nor disagreed) toward physical barriers but visitors exposed to the physical barrier, signage or a combination of both, agreed more that physical barriers improve visitor experience and penguin welfare (Table 5). Therefore, visitors in the treatment groups had more positive attitudes toward “Physical barriers” compared to visitors exposed to standard zoo conditions (Table 5).

No significant treatment effects were found on any other attitude scales or the questions where visitors rated the welfare of the penguins, the penguin enclosure and their own experience at the exhibit (*p* > 0.05).

## Discussion

Several visitor attitude scales were found to be correlated with penguin behavior, but it should be noted that due to the large number of statistical tests, only those attitude variables that were consistently correlated with more than one penguin behavior variable are discussed. In contrast, there were only a few treatment effects on these scales. The correlations indicate that the more visible, active and close the penguins were to the visitor viewing area, the more positive visitor attitudes were toward positive little penguin characteristics, penguin welfare, visitor effects, the enclosure, learning, visitor experience and exhibit manipulations. This suggests penguins that display fewer behaviors indicative of fear such as avoidance, huddling and vigilance and more behaviors that are active such as swimming and diving, elicit more positive visitor attitudes toward the penguins, their welfare, enclosure and visitor experience. Our findings are supported by studies that have found zoo animals that engage in active behaviors and increased behavioral diversity, improve visitor perceptions of the animals (Anderson et al., 2003), predict visitors’ self-reported positive affective responses (Luebke et al., 2016) and increase conservation intent (Hacker and Miller, 2016). In contrast, other studies have found zoo animals that display stereotypic behaviors such as pacing, reduced visitor perceptions of the animals’ welfare and the level of care for the animals and decreased support for zoos (Miller, 2012; Godinez et al., 2013). Thus, the current results, consistent with previous research, provides evidence that zoo animal behavior is an important factor that is associated with zoo visitor attitudes and experience.

It is well understood that human attitudes can be a strong predictor of human behavior as demonstrated by the agricultural research on human-animal relationships (Fishbein and Ajzen, 2010; Hemsworth and Coleman, 2011). Positive attitudes in stockpeople toward animals they work with, have been found to result in increased positive handling toward animals and subsequently, positive effects on animal behavior and welfare which reinforces positive handling and attitudes (Hemsworth and Coleman, 2011). Chiew et al. (2019) found similar results to that of Sherwen et al. (2015b) where the close proximity of visitors which increased intense visitor behaviors such as leaning over the enclosure, sudden movement and tactile contact with the enclosure and pool’s water, increased little penguin avoidance behavior and other behaviors indicative of fear but not fecal glucocorticoid metabolite concentrations (Chiew et al., 2019). This suggests that despite the positive visitor attitudes toward little penguins at Melbourne Zoo, visitors still had a negative effect on the penguins which contrasts with the agricultural research on human-animal relationships (Hemsworth and Coleman, 2011). This may be because positive visitor attitudes toward penguins may have increased visitors’ desire to interact or be in close contact with penguins, thus engaging in potentially intense and threatening visitor behaviors and resulting in negative effects on the penguins. However, we were not able to directly correlate each visitor’s attitudes with their behavior and in the present study we examined the general attitudes of visitors toward little penguins rather than the visitors’ attitudes specifically toward the behaviors that they, as visitors, engage in toward little penguins. Consequently, further research is clearly required to understand visitor attitudes toward the behaviors they engage in when viewing zoo animals.

It is also possible that visitors may lack knowledge or awareness of the effect they can have on zoo animals. This is supported by the finding where attitudes toward “Positive visitor effects” and “Neutral visitor effects” were on average neutral (i.e., neither agreed nor disagreed). These results suggest the uncertainty visitors have about whether little penguins find visitors positive, negative or neutral. If visitors are not aware that their behavior may result in negative consequences on penguins, provision of such information may allow visitors to choose to change their behavior that may minimize their negative effect on penguins. Abraham and Denford (2017) argue that the provision of information may be vital in changing people’s behavior when people lack an understanding of their own behavior or its consequences. Thus, visitor education to raise awareness of visitor effects may be required to shift and modify visitor behavior to minimize negative effects on zoo animals. Research in agriculture has demonstrated that stockperson attitudes and their behavior toward animals can be improved through training (Hemsworth et al., 1994; Coleman et al., 2000). Consequently, further research is required to examine visitor attitudes and behavior in conjunction with the examination of visitor effects, identifying what behaviors visitors are performing that may affect zoo animals and attitudes toward those behaviors so that they can be targeted and modified.

Despite the growing research investigating how zoo animal behavior influences visitors’ attitudes, there is still limited research to link this understanding with observations of visitor effects on zoo animals. This is important as it may help with identifying strategies to manage zoo visitor-animal interactions. For example, Blaney and Wells (2004) found that visual contact with visitors resulted in increased intra-group aggression and abnormal behaviors including repetitive teeth clenching and body rocking in gorillas (Blaney and Wells, 2004). However, installation of camouflage netting to the viewing area of the gorilla exhibit to reduce the visibility of visitors, reduced conspecific-directed aggression and stereotypic behaviors in the gorillas but also increased visitor perceptions of gorillas where they were perceived as more exciting and less aggressive (Blaney and Wells, 2004). This demonstrates that the camouflage netting is a highly suitable management strategy to manage zoo visitor-gorilla interactions that has no detrimental impact, and rather positive effect, on the animals and visitors. In contrast, some research has found that modification of zoo visitor-animal interactions using visual or physical barriers, for example, may affect visitor experience and potentially visitor attitudes despite the improvement in animal welfare (Sherwen et al., 2015a; Chiew et al., 2019). This highlights the importance of examining visitor attitudes when investigating the effects of visitors on zoo animals to identify suitable ways to manage visitor-animal interactions. Consequently, the second aim of our present experiment was to address this by determining the effects of regulating visitor-penguin interactions by imposing exhibit manipulations (i.e., treatments: physical barrier and/or signage) to the visitor viewing area on visitor attitudes.

No treatment effects were found on visitor attitudes toward penguin welfare, the exhibit, learning, visitor experience, visitor interests and visual barriers as well as how visitors rated the penguins’ welfare, the enclosure and their own experience at the enclosure. This suggests that there was no detrimental impact of a physical barrier and/or signage on these visitor attitude scales or visitor experience. Interestingly, this contrasts with the few studies that have suggested one-way visual barriers to reduce visual contact with visitors and a physical barrier to regulate visitor viewing proximity and behavior, may negatively affect visitors and their experience due to the reduced visitor numbers and reduced interaction with zoo animals at the exhibit when these barriers are in place (Sherwen et al., 2015a; Chiew et al., 2019). However, there were some differences in visitor attitudes between visitors that were exposed to standard zoo conditions, a physical barrier (set up 2 m from the enclosure), signage or a combination of both a physical barrier and signs for attitudes toward “Positive penguin characteristics,” “Neutral visitor effects” and “Physical barriers.”

Visitors exposed to standard zoo conditions had more positive attitudes that penguins are not affected by visitors compared to visitors exposed to the exhibit manipulations which on average were neutral (i.e., neither agreed nor disagreed). Considering there is evidence indicating penguins can be negatively affected by visitors (Ozella et al., 2014; Sherwen et al., 2015b; Chiew et al., 2019), this result may be a concern for zoos as it suggests that visitors exposed to standard zoo conditions have misconceptions that visitors do not affect penguins. In comparison, visitors exposed to exhibit manipulations may have considered more the potential effects visitors have on penguins because of the presence of the exhibit manipulations. Thus, this suggests that exhibit manipulations may be a positive influence on visitor attitudes toward visitor effects. However, attitudes toward “Positive little penguin characteristics” differed between visitors that were exposed only to either a physical barrier or signage, indicating visitors exposed to a physical barrier had more positive attitudes toward “Positive little penguin characteristics” compared to visitors exposed to signs. This was also found for attitudes toward “Neutral visitor effects” indicating visitors exposed to a physical barrier had slightly more positive attitudes compared to visitors exposed to signs. This suggests that the type of exhibit manipulation or strategy to manage visitor-animal interaction is important where signs may have more of a negative influence on visitor attitudes compared to a physical barrier. This is somewhat consistent with Blaney and Wells (2004) which as previously discussed found camouflage netting (i.e., a physical barrier) installed to the viewing area of the gorilla exhibit, increased positive perceptions of gorillas. However, Meis and Kashima (2017) found that what influences the perceived effectiveness of a sign is the clarity of the signs purpose, especially for unfamiliar signs which in our study were unfamiliar and may not have had a clear purpose for visitors. This could explain why there was a potential negative effect on attitudes when visitors were exposed to signs in the present study compared to visitors that were not, since limited explanation was given to visitors as to why they were requested to be quiet, move slowly and not interact with the animals. However, clearly further research is still required to understand the effectiveness of signs within zoos on visitor attitudes and behavior.

Based on the few treatment effects on visitor attitudes, the results suggest that, if a management strategy were to be implemented to manage visitor-penguin interactions, a physical barrier may be more suitable over the use of signage, having less of a negative influence on visitor attitudes compared to signs. This is also supported by our finding that irrespective of whether it was the visitors exposed to a physical barrier, signage or a combination of both, in comparison to the visitors exposed to standard zoo condition, visitor attitudes toward physical barriers were more positive. In other words, there was more agreement that physical barriers would improve both visitor experience and penguin welfare when visitors were exposed to the exhibit manipulations compared to those that were not. Furthermore, Chiew et al. (2019) found that the physical barrier reduced potentially threatening visitor behaviors such as banging on enclosure features, leaning over the pool, tactile contact with the pool’s water and sudden movement while signs had no effect on visitor behavior. This is also supported by Park et al. (2008) that found direct management by using a physical fence, was the most effective strategy to control visitor behavior compared to educational signage at Acadia National Park, United States. Consequently, our findings suggest that a physical barrier could be a suitable management strategy to manage visitor-penguin interactions. However, it should be noted that it is unclear if these few treatment effects on visitor attitudes affected visitor attitudes directly, or was a consequence of the treatment effects on penguin behavior that influenced visitor attitudes. For example, it was likely that the increased positive perceptions of the gorillas by visitors found by Blaney and Wells (2004) was influenced by the presence of the camouflage netting but also the changes in gorilla behavior because of the camouflage netting reducing visual contact with visitors.

We recognize that the methodology used in the present study, does not allow us to disentangle the direct effects on visitor attitudes of regulating visitor viewing proximity and behavior using a physical barrier and/or signage *per se*, from the effects of changes in penguin behavior on attitudes arising from this regulation. Also, the generalizability of our findings to other zoos is limited and the questionnaires completed were biased toward people living in Australia, pet owners and females which are common biases found in survey data (Driscoll, 1992; Kendall et al., 2006). Therefore, the visitors surveyed within our present study may not be representative of the population of visitors to Melbourne Zoo. Furthermore, we recognize that using the average daily penguin behavior and survey data, may have diluted the effects and masked the variation that is possible throughout the day in both penguin behavior and visitor attitudes. However, using daily averages and a randomized factorial design with three replicates of each treatment helps average out chance variation. Despite these limitations, the results gathered in our experiment provides insight on current visitor attitudes at Melbourne Zoo and has identified some influencing factors on visitor attitudes which provides a foundation for further research to build upon.

## Conclusion

This study is the first study, to our knowledge, that provides information on visitor attitudes specifically toward zoo-housed little penguins, their welfare, enclosure, visitor effects, visitor experience and exhibit manipulations at an Australian zoo. We were able to identify two factors that influence visitor attitudes which were little penguin behavior and exhibit manipulations. The more visible, active and close the penguins were to the visitor viewing area, the more positive visitor attitudes were toward positive little penguin characteristics, penguin welfare, visitor effects, the enclosure, learning, visitor experience and exhibit manipulations. However, there were limited effects of the exhibit manipulations on visitor attitudes and experience. These findings have increased our understanding of the multifaceted nature of visitor attitudes and have identified some influencing factors on attitudes that can be used to inform the way zoos manage visitor-penguin interactions, but clearly further research is required.

## Data Availability Statement

The datasets generated for this study are available on request to the corresponding author.

## Ethics Statement

This study, which involved human participants to part take in an anonymous questionnaire, received human ethics approval from the Veterinary and Agricultural Sciences Human Ethics Advisory Group, Faculty of Veterinary and Agricultural Sciences, University of Melbourne, Australia (Ethics Application 1545739.1). The participants provided their written informed consent to participate in the study.

## Author Contributions

All authors designed the study. SC was responsible for liaising with the Wild Seas keeping team and other staff at Melbourne Zoo (Zoos Victoria, Australia) as well as the staff and student volunteers and interns from the Animal Welfare Science Centre (University of Melbourne, Australia), organized and carried out the data collection with the help of student volunteers and interns from the Animal Welfare Science Centre. SC collated all data and with the aid of GC, performed the statistical analysis of the data and interpretation. SC and GC wrote the manuscript. PH, SS, and VM provided feedback and additions to the manuscript.

## Conflict of Interest

The authors declare that the research was conducted in the absence of any commercial or financial relationships that could be construed as a potential conflict of interest.
